# Vacuum-Packed Steak Tartare: Prevalence of *Listeria monocytogenes* and Evaluation of Efficacy of Listex^TM^ P100

**DOI:** 10.3390/foods11040533

**Published:** 2022-02-12

**Authors:** Lucie Hluchanova, Kristyna Korena, Helena Juricova

**Affiliations:** Department of Microbiology and Antimicrobial Resistance, Veterinary Research Institute, Hudcova 296/70, 621 00 Brno, Czech Republic; korena@vri.cz (K.K.); juricova@vri.cz (H.J.)

**Keywords:** *Listeria monocytogenes*, steak tartare, bacteriophage, Listex^TM^ P100

## Abstract

Steak tartare is a raw, ready-to-eat meal popular in European countries, the safety of which is often discussed due to the risk of foodborne illness. The aim of this study was to determine the prevalence of *Listeria monocytogenes* in vacuum-packed steak tartare from retailers in the Czech Republic, characterize the strains obtained by typing methods and to evaluate the efficacy of Listex^TM^ P100 against *L. monocytogenes* artificially inoculated into steak tartare samples. The prevalence of *L. monocytogenes* was 55% and 17 isolates belonging mostly to serotype 1/2a were obtained. Altogether 11 sequence types and 11 clonal complexes were assigned based on the whole genome sequencing (WGS) signifying the high diversity of *L. monocytogenes* isolates obtained. Core genome multi-locus sequence typing (cgMLST) did not confirm an epidemiological connection with human cases of listeriosis. The efficacy of Listex^TM^ P100 treatment at concentrations of 10^8^ and 10^9^ PFU/g on artificially inoculated beef steak tartare samples was not efficient. Based on the results of this study, steak tartare from retailers can be considered as a source of *L. monocytogenes* that remains a challenge to the food industry.

## 1. Introduction

Steak tartare is a ready-to-eat (RTE) meal popular in the Czech Republic as well as in other European countries. It is traditionally prepared from raw minced beef usually served with numerous ingredients such as raw egg yolk, a selection of sauces, vegetables, and spices. The fact that this meal is served raw poses a potential risk to consumers due to the growth of bacteria to unacceptable levels and, therefore, a potential source of food-borne infections such as listeriosis, salmonellosis, and campylobacteriosis [1].

*Listeria monocytogenes* is a ubiquitous, Gram-positive bacterium and important food-borne pathogen causing listeriosis, an infectious disease of humans and different animal species. It may cause slight flu-like symptoms in healthy people, however, listeriosis can generate severe symptoms such as gastroenteritis, meningitis, abortions, and neonatal infections in immunocompromised individuals [2]. Although the incidence of listeriosis is low in the general population, the high death rate associated with the illness makes it a significant public health problem [3]. The overall fatality rate for listeriosis in the European Union (EU) was 17.6% in 2019 and increased compared to 2017 and 2018 (15.6% and 13.6%, respectively). This makes listeriosis one of the most serious food-borne diseases under EU surveillance [4].

The vast majority of human cases of listeriosis are caused by the consumption of RTE products contaminated with *L. monocytogenes* and the outbreaks have been mostly associated with RTE foods of animal origin such as meat products, seafood, and dairy products [5]. *L. monocytogenes* is able to survive various stress conditions, which allows it to colonize many different environments as well as to persist over months or years on technological equipment in meat processing plants, which increases the risk of product contamination [6]. Raw meat as well as RTE meat products can be considered as an important source of *L. monocytogenes* [7,8,9]. Raw meat fulfills the conditions required for the growth and multiplication of *L. monocytogenes* and can be a source of cross-contamination of clean areas and equipment in food production and thus contaminates RTE food. Meat-processing facilities use cold storage (4 °C) to reduce the proliferation of bacteria on meat. Although this process is effective against many bacteria, it supports the growth of *L. monocytogenes* as this bacterium has the ability to survive at low temperature [2]. Additionally, it has been reported that *L. monocytogenes* can grow in vacuum packages as well as in modified atmosphere packages which extend the shelf-life of foods, giving the opportunity for *L. monocytogenes* to multiply to high numbers towards the end of shelf-life, especially if the recommended temperature during storage is not maintained [10].

Although raw meat is usually sufficiently heat-treated before consumption, which should eliminate *L. monocytogenes,* we can also encounter the consumption of insufficiently heat-treated meat, for example steaks, roast beef, or steak tartare. According to the European legislation (Reg. EC 2073/2005) [11], steak tartare must comply with the limit of 100 CFU/g throughout the whole shelf-life, but this criterion can only be applied if the food business operator is able to demonstrate, to the satisfaction of the competent authority, that the product will not exceed this limit until the expiry date. Other countries (i.e., USA) apply a zero-tolerance policy and require the complete absence of *L. monocytogenes* in 25 g of foodstuff. This restrictive policy implies the need for improving hygiene procedures to prevent contamination from this common bacterial agent [12].

A promising approach to prevent or decrease the occurrence and persistence of *L. monocytogenes* is the use of a bacteriophage. A phage that is commonly used to combat *L. monocytogenes* is P100 [13]. This phage was originally isolated from the wastewater of a dairy plant and is not considered to pose a risk to human health because of the lack of toxicity in rats and because it is strictly lytic to bacteria while being unable to transduce bacterial DNA. It is commercially available as Listex^TM^ P100 [14].

The aim of this study was (i) to determine the prevalence of *L. monocytogenes* in samples of vacuum-packed steak tartare from retailers in the Czech Republic, (ii) to characterize the strains obtained, and (iii) to perform a model experiment determining the efficacy of Listex^TM^ P100 against *L. monocytogenes* artificially inoculated into steak tartare samples.

## 2. Materials and Methods

### 2.1. Prevalence and Characteristics of L. monocytogenes from Steak Tartare Samples

Sample collection: A total of 20 samples of vacuum-packed beef steak tartare from two producers in the Czech Republic (*n* = 9) and two producers in Poland (*n* = 11) were obtained in the period 2017–2020 from Czech retailers. All samples were maintained at a temperature not exceeding 4 °C during transport and storage and were analyzed at the end of the expiry date. The samples of steak tartare were purchased according to current availability to retail customers.

Isolation of *L. monocytogenes*: Enumeration of *L. monocytogenes* in the samples was carried out according to the International Organization for Standardization (ISO) methods EN ISO 11290–2:2017. Culture method of *L. monocytogenes* was performed according to EN ISO 11290–1:2017 [15]. Briefly, 25 g of each sample was aseptically weighed and homogenized with 225 mL of half-Fraser broth (Oxoid Ltd., Basingstoke, United Kingdom) and incubated at 30 °C/24 h for primary enrichment. Thereafter, 0.1 mL of the culture obtained was inoculated into 10 mL of Fraser broth (Oxoid Ltd., Basingstoke, United Kingdom) and incubated for 24 h at 37 °C. After the enrichment, COMPASS Listeria Agar (BIOKAR Diagnostics, Allonne, France) and Rapid´L.mono (Bio-Rad Laboratories, Inc., Hercules, CA, USA) were used for selective plating. After incubation for 48 h at 37°C the colonies of *L. monocytogenes* were confirmed based on morphological properties.

Serotyping: The strains obtained were serotyped by multiplex polymerase chain reaction (PCR) according to Doumith et al. [16] in combination with rapid slide agglutination using commercially available antisera (Denka Seiken Co. Ltd., Tokyo, Japan) according to the manufacturer’s instructions.

Whole genome sequencing: Genomic DNA was extracted using the Blood and Tissue kit according to the manufacturer’s instructions (Qiagen, Hilden, Germany). Preparation of DNA libraries by Nextera XT DNA Library Preparation Kit (Illumina, Inc., San Diego, CA, USA) and sequencing on the Illumina platform were carried out externally using Illumina NextSeq sequencers (LGC Genomics GmbH, Berlin, Germany).

Genome assembly and core genome multi-locus sequence typing (cgMLST): Genome assembly, cgMLST, and data analysis was performed by using Ridom SeqSphere+ software (version 7.2.0; Ridom GmbH, Münster, Germany). The resulting FASTQ files were first quality trimmed and then de novo assembled using Velvet assembler integrated into Ridom SeqSphere+ software. Isolates were compared in seven MLST and 1701 cgMLST targets [17]. To compare the relationship between isolates from steak tartare and human isolates using cgMLST, the sequences of human isolates of the same sequence types (ST) were selected from the Veterinary Research Institute collection from the period 2017–2021.

### 2.2. Model Experiment Using Listex^TM^ P100

Sensitivity testing of *L. monocytogenes* strains to Listex^TM^ P100: The Listex^TM^ P100 stock solution (Micreos Food Safety, Wageningen, Netherlands) at a concentration of 10^11^ PFU/mL was used. Serial dilutions from 10^11^ to 10^5^ PFU/mL were used to assess susceptibility of a total 58 *L. monocytogenes* strains (data not shown). Based on the results, isolates showing the highest level of sensitivity (LV1242) and resistance (LV830) to Listex^TM^ P100 were selected for further analysis.

Efficacy of Listex^TM^ P100 on reduction of *L. monocytogenes*: The efficacy of Listex^TM^ P100 against *L. monocytogenes* was tested in two independent experiments with two concentrations of Listex^TM^ P100 bacteriophage. In each experiment, 24 samples of steak tartare, 10 grams each were prepared. Half of the samples (*n* = 12) were artificially inoculated with a sensitive isolate of *L. monocytogenes* LV1242, and the remaining half of the samples were inoculated with a resistant isolate of *L. monocytogenes* LV830 to a final concentration of 10^3^ CFU/g. Samples were divided and subsequently, Listex^TM^ P100 was added to six samples inoculated with the LV1242 sensitive strain and/or LV830 resistant strain. The remaining samples without Listex^TM^ P100 controlled for the growth potential of *L. monocytogenes* in steak tartare samples. In the first experiment, 10 µL Listex^TM^ P100 was added to achieve a final concentration of 10^8^ PFU/g. In the second experiment, 100 µL Listex^TM^ P100 was added to achieve a final concentration of 10^9^ PFU/g. The samples were kept at 4 °C. Immediately after inoculation and 1, 5, 24, 48, and 72 h after inoculation, 90 mL peptone water was added to 10 g of steak tartare, then homogenized and the counts of *L. monocytogenes* were established, as described above. Simultaneously, experiments with the same arrangements were performed in TSB (Tryptone Soya Broth, Oxoid Ltd., Basingstoke, United Kingdom) for comparison.

## 3. Results and Discussion

### 3.1. Prevalence and Characteristics of L. monocytogenes from Steak Tartare Samples

A total of 20 samples of vacuum-packed beef steak tartare from four producers (Czech Republic and Poland) were analyzed. *L. monocytogenes* was detected in 11 samples out of 20 from all tested producers, which indicates a prevalence of 55%. Altogether, 17 *L. monocytogenes* isolates were obtained since more than one isolate of *L. monocytogenes* was detected in five samples of steak tartare based on the results of molecular typing. The prevalence of *L. monocytogenes* reported in raw meat products is often higher than that of RTE meat products [2,18,19]. Due to the fact that steak tartare is raw minced beef meat and RTE food at the same time, the prevalence of both commodities was compared. In previous studies, *L. monocytogenes* was found in raw minced beef with a prevalence from 7.2% to 52% [18,19,20]. According to European Food Safety Authority (EFSA) and European Centre for Disease Prevention and Control (ECDC), 26 member states (MS) of the European Union (EU) and three non-MS reported data from RTE meat products over the 2016–2019 period with the overall prevalence of *L. monocytogenes* at 2.9% (1634 positives out of 56,070). Specifically, RTE products from beef meat analyzed in 2019 were positive for *L. monocytogenes* in 2.8% of the 2038 units tested in the EU [4]. *L. monocytogenes* prevalence found in our study showed higher contamination percentages compared to the EU prevalence in RTE meat product as well as in raw minced beef meat. However, enumeration of *L. monocytogenes* in steak tartare resulted in no product exceeding the limit of 100 CFU/g at the end of shelf-life and was in compliance with the EU food safety criterion. In comparison, 0.65% out of 2295 RTE meat product tested samples from 12 MS of the EU in 2019 were found to exceed the criterion of 100 CFU/g [4]. For a healthy human population, food products with levels below the limit of 100 CFU/g limit are considered to pose a very low or negligible risk [10]. Nevertheless, even a product with a low-level of *L. monocytogenes* contamination could represent a risk of severe illness for immunocompromised individuals [21].

Molecular serotyping performed by PCR in combination with rapid slide agglutination revealed that the majority of isolates obtained from steak tartare samples belonged to 1/2a serotype (12/17; 71%), followed by 1/2b (2/17; 12%), 1/2c (2/17; 12%), and 4b (1/17; 6%). In one sample of steak tartare from a Czech producer, isolates belonging to three different serotypes (4b, 1/2b, 1/2c) were confirmed. The dominance of serotype 1/2a in RTE meat products and raw meat was reported also in previous studies [22,23]. For example, in Estonia, 51% and 47% of strains belonged to serotype 1/2a originating from RTE meat products and raw meat, respectively [24]. Several studies showed that most human listeriosis outbreaks have been associated with *L. monocytogenes* serotype 4b. The largest documented outbreak of listeriosis in South Africa occurred between 2017 and 2018 and was associated with the consumption of polony, which is a RTE meat product, and was of *L.* *monocytogenes* of serotype 4b [2]. However, serotype 1/2a was the most frequent serotype associated with human cases of listeriosis in the Czech Republic between 2013 and 2020 [25].

**Figure 1:**
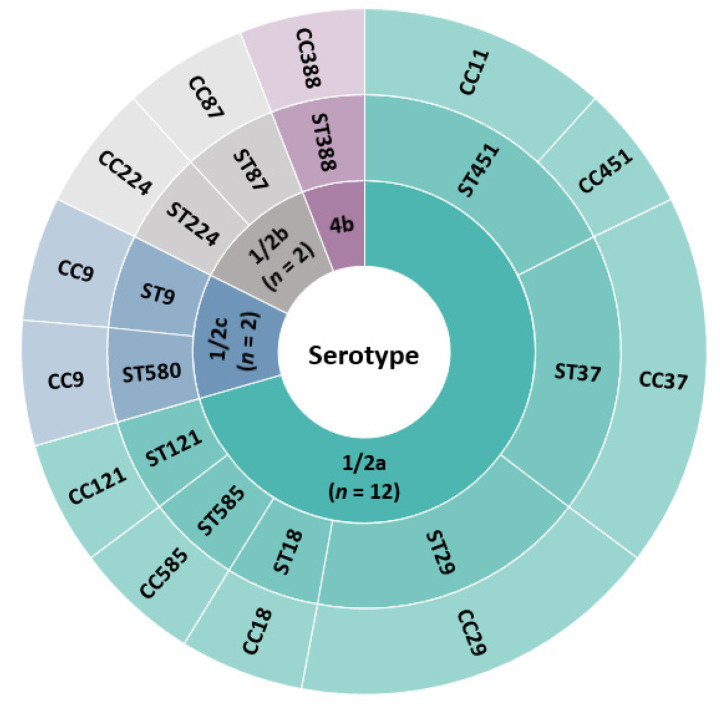
Graphical representation of the diversity of *L. monocytogenes* strains obtained from steak tartare samples.

**Figure 2:**
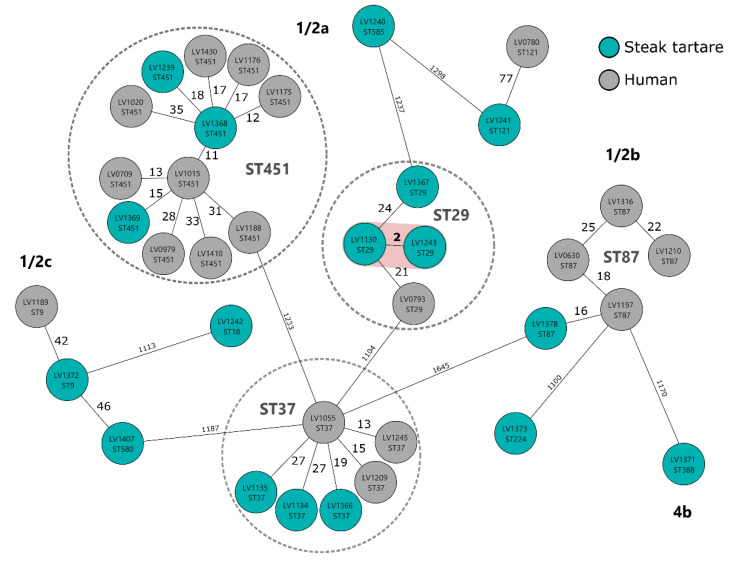
A minimum spanning tree (MST) showing the core genome allelic diversity of *L. monocytogenes* isolates from steak tartare (green nodes) and human cases of listeriosis (gray nodes). The LV numbers inside the nodes represent the laboratory identification of the strains. The MST is based on 1701 cgMLST loci. Numbers on the depicted branches represent the absolute distance between genotypes in the number of loci. The clusters where the distance between at least two isolates is 10 alleles or less are highlighted.

Using whole genome data, isolates were assigned to 11 sequence types and 11 clonal complexes (Figure 1) suggesting a high variability of isolates. Sequence types ST29 (CC29, *n* = 3), ST37 (CC37, *n* = 3), and ST451 (CC11, *n* = 2; CC451, *n* = 1) were found to be the most frequent. The appearance of ST29, ST37, and ST451 was recently described in connection with cattle abortion cases in Latvia during 2013–2018 as well as in a study on the genetic diversity of *L. monocytogenes* from dairy production in the United States [26,27]. This may suggest that these particular STs are often associated with cattle. In the Czech Republic, ST451 has also been described in association with rabbit meat, with all isolates obtained in 2013–2016 belonging to serotype 1/2a ST451, which may indicate the presence of a potentially persistent strain in a rabbit meat processing plant [28]. The ability to persist has been described especially in *L. monocytogenes* ST121 strains [29]. Only one isolate of ST121 was detected in our study. Since 2017, 19 cases of listeriosis in the Czech Republic have been caused by strains of *L. monocytogenes* belonging to the same STs (ST451, ST37, ST29, ST87, ST9, ST121) as those identified for isolates from steak tartare samples [25]. Therefore, the genomes of human and steak tartare isolates were compared based on cgMLST (Figure 2).

Core genome MLST analysis revealed only one cluster with between-isolate distance less than 10 alleles, which is a standard cutoff for *L. monocytogenes* cgMLST-based epidemiological analysis. Two isolates of *L. monocytogenes* ST29 from two different batches of steak tartare samples from one producer differed in only two alleles. These two samples were produced one year apart, which may indicate a potential persistent strain in the production plant environment or reintroduction of a strain, e.g., from raw beef meat from the same supplier. As can be seen in the MST, most of the strains originating from steak tartare samples and human cases of listeriosis were not closely related to each other (>10 alleles). However, in the ST451 group, the allelic difference between isolates originating from steak tartare and humans closely exceed the above-mentioned limit of 10 alleles. This may indicate a high genetic relatedness in ST451 strains, but it cannot be considered as a source of infection in this case. In this study, we demonstrated a relatively high diversity of *L. monocytogenes* isolates obtained from packed steak tartare from retailers and these isolates do not appear to be epidemiologically connected with human isolates.

### 3.2. Model Experiment Using Listex^TM^ P100

The use of biological solutions such as Listex^TM^ P100 appears to be a promising choice to further reduce the load of undesirable bacteria including *L. monocytogenes* in food products. Therefore, we were interested in the efficacy of Listex^TM^ P100 treatment against *L. monocytogenes* in beef steak tartare samples during storage at 4 °C. *L. monocytogenes* sensitive isolate LV1242 and resistant isolate LV830 were artificially inoculated at initial concentrations of 10^3^ CFU/g into steak tartare samples and to TSB medium. Two concentrations of Listex^TM^ P100 were used for evaluation. In parallel, the samples without Listex^TM^ P100 addition served as a positive control for LV1242 and LV830 survival/growth in the samples. 

**Figure 3:**
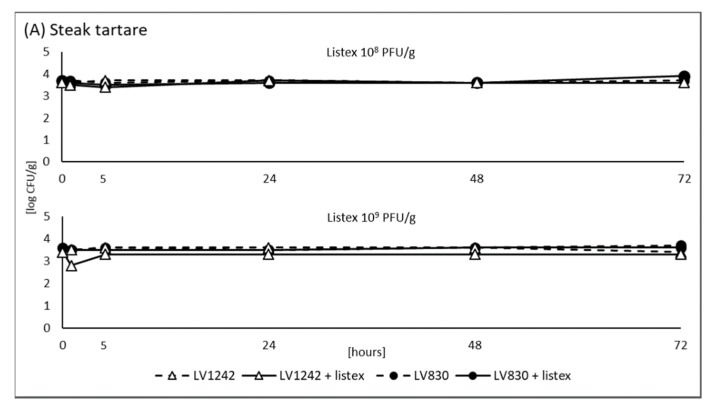

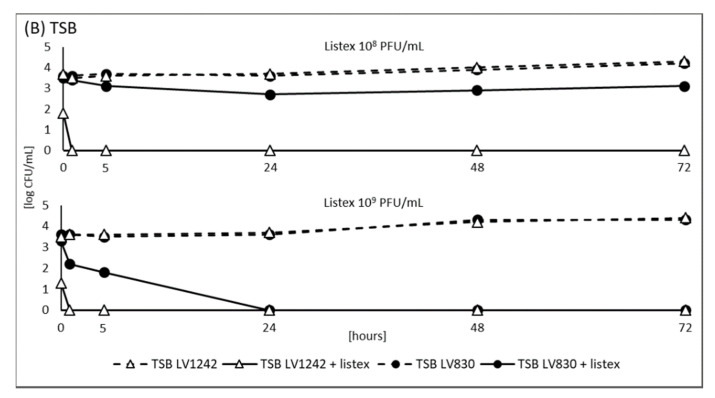
The efficacy of Listex^TM^ P100 against *L. monocytogenes* sensitive isolate LV1242 and resistant isolate LV830 in beef steak tartare samples (**A**) and in TSB (**B**) during storage at 4 °C. Dashed lines: inoculated samples of steak tartare and/or TSB without Listex^TM^ P100; solid lines: inoculated samples of steak tartare and/or TSB with Listex^TM^ P100.

In steak tartare, Listex^TM^ P100 at 10^8^ PFU/g was not effective against sensitive isolate LV1242 and resistant isolate LV830 at all (Figure 3A). By using 10^9^ PFU/g, a slight reduction in counts of LV1242 sensitive isolate was observed after 1 h of treatment, however, this reduction did not affect the total counts of LV1242 at the end of the experiment. No effect after application of 10^9^ PFU/g was seen on numbers of LV830 resistant isolate. Compared to the control, TSB medium (Figure 3B), the complete reduction of *L. monocytogenes* sensitive isolate LV1242 was achieved immediately at the time of application by both concentrations of Listex^TM^ P100. One logarithmic order reduction of LV830 resistant isolate was observed after 24 h by using 10^8^ PFU/mL. However, at the end of the experiment, the numbers of LV830 almost reached the level at inoculation. By using 10^9^ PFU/mL of Listex^TM^ P100, a complete removal of LV830 isolate was achieved after 24 h.

The effectiveness of Listex^TM^ P100 against *L. monocytogenes* in different RTE products such as raw salmon fillets, channel catfish fillets, or surface-ripened soft cheese were already described [13,30,31] and subsequently in 2016, the use of Listex^TM^ P100 was approved by EFSA [14]. According to EFSA, treatment with Listex^TM^ P100 should only be considered as an additional tool following the application of Good Hygienic Practices (GHP) and Good Manufacturing Practices (GMP). Although Listex^TM^ P100 is unable to completely eliminate *L. monocytogenes* present in a product at levels 100–10,000 CFU/g, it is expected to prevent bacterial counts from exceeding the legislative limit of 100 CFU/g [14]. Even though we were aware of this limitation, the inoculation levels of *L. monocytogenes* in model experiments were 1,000 CFU/g because lower levels of *L. monocytogenes* would be difficult to determine exactly. However, in control medium such a level of bacteria was evaluated to be eradicable. The study by EFSA also indicated that a proportion of naturally occurring *L. monocytogenes* strains exhibited resistance to bacteriophage P100. According to our preliminary results, resistant strains exist and, as has been seen, their elimination (in a suitable medium) requires more time and a higher concentration of phage.

As a method of application, surface treatment by spraying or dipping the RTE food into the bacteriophage solution is recommended [14]. The efficacy of Listex^TM^ P100 in steak tartare samples was insufficient in our model experiment. This could be explained by several factors, such as by the fact that bacterial contamination of steak tartare from minced meat can occur throughout the product, not just on its surface and thus can be less accessible for sufficient eradication. Furthermore, the possible influence of various ingredients used in the product must be taken into account. Therefore, it is obvious that *L. monocytogenes* remains a challenge to food safety of this particular item.

## 4. Conclusions

Although the number of *L. monocytogenes* in the steak tartare samples complied with the EU food safety criterion, steak tartare at the retail level can be considered as a source of *Listeria monocytogenes* and should be approached with caution. This study showed rather high diversity of *L. monocytogenes* isolated from packed steak tartare from retailers and did not confirm the epidemiological connection with human cases of listeriosis in the Czech Republic. Based on our results, Listex^TM^ P100 does not appear to be suitable for preventing bacterial contamination of *L. monocytogenes* strains in steak tartare.

## Data Availability

Not applicable.
